# Toward esthetically and biomechanically reliable anterior resin composite restorations: Current clinical experiences among dental practitioners in Saudi Arabia

**DOI:** 10.12688/f1000research.130981.1

**Published:** 2023-04-03

**Authors:** Khalid S. Almulhim, Rasha AlSheikh, Moamen Abdalla, Rasha Haridy, Amr Bugshan, Stephen Smith, Mohammed Zeeshan, Moataz Elgezawi

**Affiliations:** 1 Restorative Dental Sciences, Imam Abdulrahman Bin faisal University, Dammam, Saudi Arabia, 1982, Saudi Arabia; 2 Substitutive Dental sciences, Imam Abdulrahman Bin Faisal University, Dammam, Saudi Arabia, 1982, Saudi Arabia; 3 Clinical Dental sciences, Princess Nourah Bint Abdulrahman University, Riyadh, Saudi Arabia, Saudi Arabia; 4 Biomedical dental sciences, Imam Abdulrahman Bin Faisal University, Dammam, Saudi Arabia; 5 Preventive Dental Sciences, Imam Abdulrahman Bin Faisal University, Dammam, Saudi Arabia; 6 Medical Education, Imam Abdulrahman Bin Faisal University, Dmmam, Saudi Arabia

**Keywords:** Keywords: cross-sectional survey; resin composite; anterior teeth; general dentists; Saudi Arabia.

## Abstract

**Background:** Esthetic anterior composite restorations are very challenging. They constitute a main part of the clinical practice of restoring teeth with resin composites. Distinctive variations in composite material selection and technique of application in anterior teeth exist when compared to the practice of restoring posterior teeth with resin composites. In a continuation of a previous assessment of class II resin composite practice,  a cross-sectional survey study was performed to determine the awareness of general dental practitioners in Saudi Arabia about resin composite restorations in anterior teeth and to provide evidence-based recommendations to improve their practice.

**Methods:** A total of 500 dental practitioners from different provinces in Saudi Arabia were invited to participate in an online questionnaire which comprised four domains and included a total of sixteen questions addressing aspects regarding the selection of resin composites, cavity configuration, etching protocol, light-curing technique, liner application, application of poly-chromatic composite, as well as finishing and polishing procedures. Chi square testing and descriptive statistics were used to analyze the attained data.

**Results:** From 250 respondents, the greatest participation was from the Eastern and Middle provinces of Saudi Arabia. Female dentists participated less than males. There was no general agreement between participants regarding the resin composite material or the employed technique of application in restoring anterior tooth defects. Respondents indicated that discoloration and hypersensitivity were the most common reasons for patient’s dissatisfaction with resin composites in anterior teeth.

**Conclusions:** Dental practitioners are highly encouraged to improve their clinical practice of restoring anterior teeth using resin composites with the focus upon continuous education programs, online webinars, and workshops.

## Introduction

The main objective of the modern practice of restorative dentistry is the reestablishment of optimum esthetics and effective biofunction of hard structural tooth defects in an evidence-based and patient-centered manner with long term clinical durability. ^1^ The central focus for dental clinicians, researchers as well as patients constitutes the optimization of procedural outcomes when restoring defective anterior teeth into esthetically pleasing, biologically healthy and mechanically sound integral units of the dental arch. ^2^^–^^4^

Presently, direct resin composites are the principle and most widely used option for restoring anterior teeth as they can produce restorations with optimum esthetics and effective biomechanical rehabilitation with long-term clinical reliability and patient satisfaction. ^5^ Furthermore, they can conserve and reinforce sound tooth structure. ^6^^–^^10^ Modern resin composites have particularly upgraded optical properties, surface finish color stability, wear resistance, flexure strength and fracture toughness. ^11^ In comparison to the indirect options of dental ceramics, direct resin composites are timesaving, less costly and allow for repairable options. ^12^^–^^14^

Restoration of defects in the esthetic zone is challenging. According to World Dental Federation (FDI) commission 2-95, the quality of the restoration is directly related to the utilized materials, to the clinician's individual skills and preferences as well as to patient’s particular oral environment and post-operative care. ^2^^,^^3^ Operator related factors have been considered as being instrumental in determining the quality and clinical performance of dental restorations. This includes procedural excellence variations, clinical judgement, and differences between operators regarding cavity design, material selection and manipulation. ^15^

Various resin composite materials are currently available in the market with variations in composition, clinical performance and application technique as recommended by manufacturers. ^16^^–^^18^ Micro-filled, micro-hybrid, nano-hybrid, flowable, incremental, and bulk-fill composites are amongst the available resin composites widely used in restoring tooth defects. ^16^^–^^18^

Restoring anterior tooth shape and optical characteristics involve a variety of procedural techniques which are determined by the material used, extent of the defect and operator preference. ^19^^,^^20^ Anterior tooth defects may be small or extensive, resulting from caries or non-carious developmental defects, as well as traumatic lesions. Freehand, palatal putty index and different matricing techniques have been suggested for restoring anterior tooth defects, especially involving the incisal angle or extensive coronal tooth structure defects. ^19^^,^^20^

Natural teeth consist of translucent enamel and relatively opaque dentin which is influenced by the pulp and investing gingiva. ^21^ This necessitates a polychromatic manner of restoring anterior tooth defects requiring consideration of tooth optical characteristics and chameleon effects. ^22^^,^^23^ Individual shading patterns and translucency variability of teeth are demanding. A single shade mono-laminal versus different shade multilaminate techniques have been alternatively implemented to optimize results that will match tooth optical characteristics when restoring anterior tooth defects. ^24^^,^^25^ Dentists differ in their judgment regarding the necessity of using an intermediate liner beneath resin composites in anterior teeth. ^26^ Moreover, they differ in selecting a particular brand of resin composite material and a specific restoration technique for a particular clinical situation when restoring anterior tooth defects.

This cross-sectional survey was therefore designed to investigate the current clinical practices of general dental practitioners in Saudi Arabia regarding the restoration of anterior tooth defects using resin composites. In our previous publication, class II resin composite practices of general dental practitioners in Saudi Arabia were assessed. ^27^ The objective of the current study was to assess the knowledge and attitude of general dental practitioners in Saudi Arabia toward anterior resin composites.

## Methods

General non-university governmental hospitals in each of the following Saudi provinces; North, Middle, East, West, and South were considered in the study. The hospitals were enumerated in sequence according to the number of available dental units. The first five hospitals with the largest number of dental units in each of the study provinces with the largest number of appointed dentists were selected for the study and included 500 dental practitioners. The study was conducted over a period of four months starting mid-September 2021. E-mail addresses of general dentists were collected through administrative contacts in hospitals included in the study after explaining the study objective. Personal data including names, gender or years of employment were not available to the study investigators prior to sending the invitations, and all e-mail invitations were anonymous. Potential sources of bias were minimized by avoiding leading answers, extreme answers, questions framing, question order bias and sampling bias.

This cross-sectional study was conducted utilizing an online uploaded survey questionnaire using Google’s free online survey platform. E-mail invitations stated clearly that participation was anonymous and voluntary and that participants were free to leave the survey or to skip any questions. The targeted population of 500 resulted in a calculated sample size of 218 dentists. Calculations were performed at the 95% confidence level using a free online sample size calculator.

The validity and reliability of the questions were tested for content validation. Ten experts were invited to validate each question of the survey before the beginning of the study using the following scale: the question is not relevant, the question is somewhat relevant, the question is quite relevant, or the question is highly relevant. The experts ranked all questions as either relevant or highly relevant. Therefore, no modifications were made in the designed questions.

The questionnaire comprised four domains; the first domain focused on the demographic data of the participants. Demographic data included age, gender, and city of appointment. The second domain targeted the professional record (profession duration and frequency of utilizing anterior composite resin restorations). The third domain addressed the individual personal preferences in the selection and utilization of resin composites for restoring anterior tooth structure defects. The fourth domain investigated patients' feedback regarding previously placed resin composite restorations. Moreover, it considered suggestions for anterior resin composites practice improvement. The questionnaire included a total of 18 questions of which 3 questions allowed two optional answers while the rest of the questions included more than two optional answers. Chi square testing using SPSS 27 software at a level of significance of *p* ≤ 0.05 and descriptive statistics were employed in analyzing the received responses. Minitab statistical software is an example of free online software that can be used to perform similar statistical analysis.

**Table 1.** Hospitals targeted.

Hospital targeted	Kingdom region
Arar Central Hospital	**North**
Turaif General Hospital	
Prince Abdulaziz Bin Musaad Hospital	
Rafha Central Hospital	
Alwiqela General Hospital	
Dammam Central Hospital	**Eastern**
Qatif Central Hospital	
King Fahad Specialist Hospital	
Jubail General Hospital	
Kafji General Hospital	
General Hospital at King Saud Medical City	**Middle**
Braidah General Hospital	
AlAflaj General Hospital	
Arras General Hospital	
King Saud Hospital at Unnizah	
AlKhakeer General Hospital	**South**
King Khalid Hospital In Najran	
Najran General Hospital	
Sharourah General Hospital	
Sharourah Armforce Hospital	
King Fahad Hospital Jeddah	**West**
King Abdulaziz Hospital Makkah	
King Fahad Hospital Almaddinah	
Rabigh General Hospital	
Noor Specialist Hospital	

### Ethics statement

This research received an ethical approval (IRB-2022-02-102) from IRB Committee, Imam Abdulrahman Bin Faisal University.

During the research, participants were invited to join the survey through emails that clarified the objectives of the study and included necessary information and that joining the survey is anonymous and voluntary. The invite explained that by completing the survey, they were consenting to participate in the study. Participants were informed that they could leave the study at any step if they did not wish to continue, and that the data collected would be used for research purposes. The IRB approval of Imam Abdulrahman bin Faisal University deemed this method of implied consent as sufficient.

## Results

**Figure 1.**
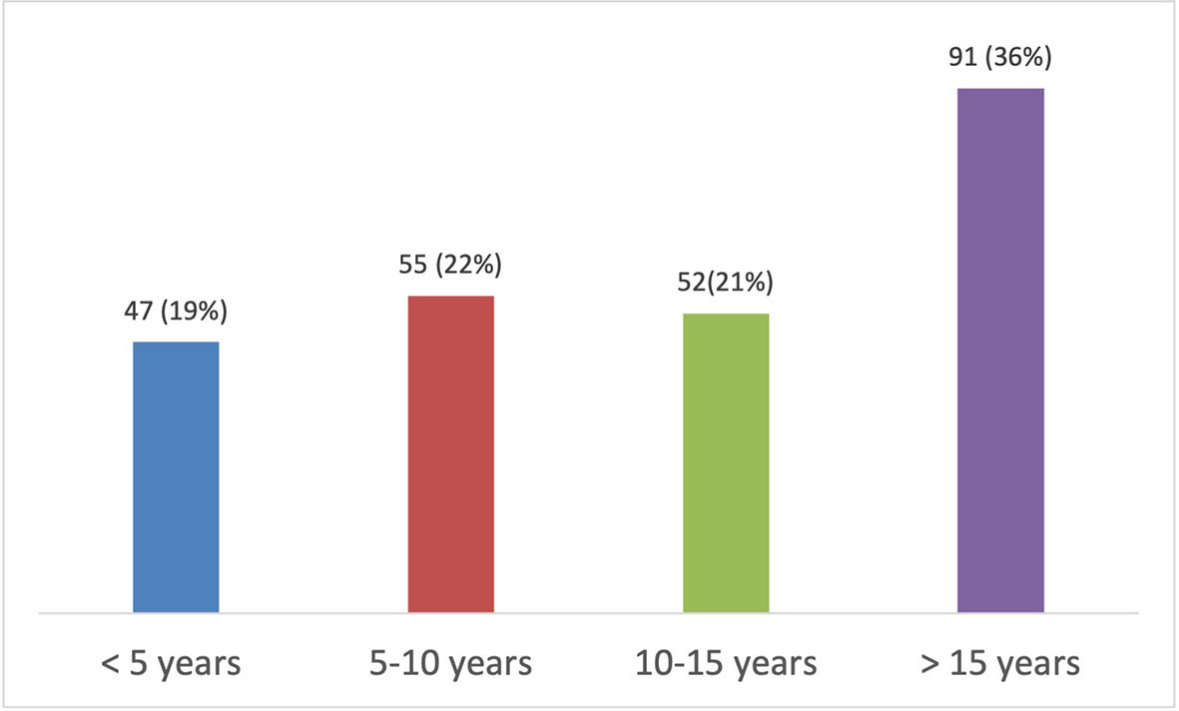
Duration of practice of dentistry.

**Figure 2.**
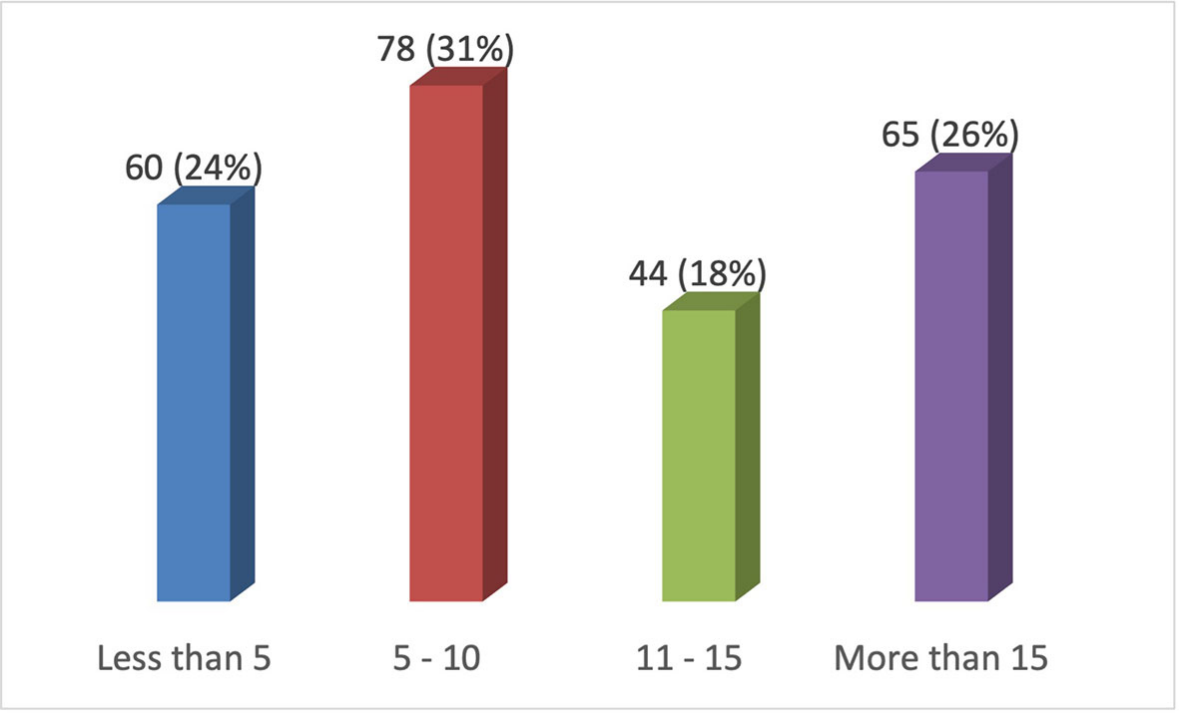
Frequency of practice of resin composite restorations in anterior teeth per week.

Out of the 500 email invitations, 250 responses were obtained. The questionnaire domains, questions, answers, and relative responses in percentages and the P values are depicted in Tables 2- 4 and Figures 1- 2: Responses with different upper-case letters in each question have statistically significant difference ( *p* ≤ 0.05). Domain 1 comprised the demographics of participants (n=250), see Table 2.

**Table 2.** Demographics of partcipants.

Questions	Modalities	Respondent	Response in %
1. Age	23-30	47	19
	31-39	101	40
	40-49	69	28
	Above 50	32	13
	**Non-respondents**	1	0
2. Gender	Male	169	68
	Female	80	32
	**Non-respondents**	1	0
3. Status	General dentist	171	68
	Resident	19	8
	Specialist	48	19
	Consultant	9	4
	**Non-respondents**	3	1
4. Where you have been practicing in Saudi Arabia	Eastern province	79	32
	Western province	55	22
	Middle province	68	27
	Northern province	21	8
	Southern province	22	9
	**Non-respondents**	5	2

Domain 2 comprised the dentists’ professional records (profession duration and frequency of utilizing anterior composite resin restorations).

**Table:** 

Questions	Modalities	Respondent	Response in %
5. How long have you been practicing dentistry? (n=250)	**<5**	47	19
	**5–10**	55	22
	**10–15**	52	21
	**15<**	91	36

6. How many resin composite restorations in anterior teeth do you place per week? (n=250)

Domain 3 comprised the individual personal preferences in the selection and utilization of resin composites for restoring anterior tooth structure defects (n=250), Table 3.

**Table 3.** Personal Preference for anterior resin Composite restorations.

Questions		Modalities	Respondent	Percentages	P-values
7. What is the isolation technique that you usually use?		Rubber dam isolation	180	73 ^a^	< 0.001
		Cotton rolls isolation	67	27 ^b^	
8. What is the most frequent type of composite resin restoration you practice?		Class III	161	64 ^a^	<0.001
		Class IV	116	46 ^a^	
		Class V	147	59 ^a^	
		Buildup	120	48 ^a^	
		Laminate	27	11 ^b^	
		Other	18	7 ^b^	
9. What is the type of resin composite material you use to restore each class?	**Class V**	Micro-filled	69	28 ^a^	0.02
		Micro-hybrid	40	16 ^a^	
		Flowable	64	26 ^a^	
		Nano-hybrid	70	28 ^a^	
		Any composite	6	2 ^b^	
		**Non-respondents**	1	0 ^b^	
	**Class III**	Micro-filled	68	27 ^a^	0.019
		Micro-hybrid	80	32 ^a^	
		Nano-hybrid	91	37 ^a^	
		Any composite	8	3 ^b^	
		**Non-respondents**	3	1 ^b^	
	**Class IV**	Micro-filled	65	26 ^a^	<0.001
		Micro-hybrid	83	33 ^a^	
		Nano-hybrid	82	33 ^a^	
		Other	14	6 ^b^	
		**Non-respondents**	6	2 ^b^	
10. Type of etching/adhesive system you use:		Self-adhesive	58	23 ^a^	<0.001
		(selective) Self-etching	78	31 ^a^	
		Total etching	112	45 ^a^	
		**Non-respondents**	2	1 ^b^	
11. Type of light-curing unit you use:		QTH (quarz Tungsten halogen)	80	32 ^a^	<0.001
		LED Blue light	161	65 ^a^	
		Laser	3	1 ^b^	
		Plasma Arch	5	2 ^b^	
		**Non-respondents**	1	0 ^b^	
12. Duration of curing per layer:		10 sec.	43	17 ^a^	<0.001
		20 sec.	136	54 ^a^	
		30 sec.	37	15 ^a^	
		40 sec.	27	11 ^a^	
		More than 40 sec.	4	2 ^b^	
		**Non-respondents**	3	1 ^b^	
13. In class IV restorations, what is the technique that you use?		Freehand technique	108	43 ^a^	<0.001
		Palatal putty index technique	26	10 ^a^	
		Both alternatives	114	46 ^a^	
		**Non-respondents**	2	1 ^b^	
14. Do you usually use a liner under a composite restoration?		No	27	11 ^a^	<0.001
		Sometimes	138	55 ^a^	
		Most of the times	83	33 ^a^	
		**Non-respondents**	2	1 ^b^	
15. In class IV restorations, do you use a polychromatic approach in the form of enamel and dentin shades?		Yes	125	50 ^a^	<0.001
		No	120	48 ^a^	
		**Non-respondents**	5	2	
16. The technique used for finishing and polishing of the composite resin restoration		Soflex	82	33 ^a^	0.01
		White stones	133	53 ^a^	
		Finishing carbide burs	117	47 ^a^	
		Polishing rubber burs with paste (e.g., enhance)	118	47 ^a^	
		Finishing/Polishing strips	173	69 ^a^	
		**Non-respondents**	2	1 ^b^	

Domain 4 comprised anterior composites failure & suggestion for practice improvement (n=250) Table 4.

**Table 4.** Failure of anterior composites & Suggestion for practice improvement.

Questions	Modalities	Respondent	Response in %	
17. What are the principal forms of failure and patient dissatisfaction with old resin composite restorations in anterior teeth?	Hypersensitivity	17	7 ^b^	<0.001
	Discoloration	183	73 ^a^	
	Recurrent caries	27	11 ^b^	
	Shape and form	8	3 ^b^	
	Chipped/Fractured	13	5 ^b^	
	**Non-respondents**	2	1 ^b^	
18. How can the practice of anterior composites be improved?	Continuous education programs workshops, online webinars	170	69 ^a^	<0.001
	Direct clinical training at respective hospitals and assisting senior specialist	78	31 ^a^	
	**Non-respondents**	2	1 ^b^	

## Discussion

This study analyzed the current practice of restoring anterior tooth defects with direct resin composites by dentists in Saudi Arabia. This cross-sectional survey was utilized to collect and analyze dental practitioners’ responses at one single time. The study was designed and conducted over a period of four months which was initiated in mid-September 2021. The number of anticipated participants at the time of the study design as well as the number of respondents to the survey could have affected the results of the survey. A broader study outcome could have been provided if greater population sizes and larger numbers of respondents were utilized. ^28^^,^^29^ The inclusion of general governmental hospitals granting dental treatments completely free of charge to patients is another possible limitation of the current study. The flow of patients seeking free dental treatments versus the availability of time and facilities may also have had an influence. Study outcomes in this study could have been expanded by the addition of private dental centers and clinics as well as university clinics and hospitals. This would thus have included general dentists exposed to socioeconomically different patients and distinctive facilities. The current survey was conducted as an online questionnaire. This method facilitated the ease of collecting data instead of utilizing a physical paper method. Invitations to participants was sent via e-mail. E-mail addresses of the general dentists in the included hospitals were anonymously collected through administrative contacts after explaining the study objective. Participants were anonymously and voluntarily invited to participate in the survey without any bias and with assurance of confidentiality so as to follow ethical guidelines. To assure reliability and validity of questions, ten experts in restorative dentistry were requested to validate the questionnaire before starting the survey. All invited experts validated questions as either highly relevant or relevant. ^28^ Participants were from all Saudi Arabia provinces, with the highest participation from the Eastern and Middle provinces. Though it was not anticipated for gender to have any impact on the collected data, it was included in the general demographic information. Most of the dental schools in the Kingdom are segregated with more male schools available compared to female’s, that said, more female dentists have graduated from King Saud University since 1982 ^30^ and later from other universities with the number continuing to increase. The results from our study showed that male dentist’s participation was more than females which is in line with a previous study. ^27^

Data from the Saudi Commission for Health Specialties in 2016 shows that 70.27 % of licensed dentists are registered as general dentists in Saudi Arabia, therefore the current study design targeted general dentists who constitute the greatest fraction of licensed dentists in Saudi Arabia. ^27^ The used population size and distribution in the current study was previously used in a cross-sectional survey that studied class II resin composites practice of general dentists in Saudi Arabia. ^31^

In a former survey-based study performed in southern Brazil, a sample size of 276 dentists and descriptive statistics were used to draw the study conclusions about the clinical practice of general dentists in anterior composite restorations. ^3^ The study revealed that most dentists in southern Brazil use hybrid composite, simplified adhesives, LED light curing and immediate polishing while only few dentists use of rubber dam isolation. ^3^

A recent systematic review that studied the clinical reliability of anterior composites has summarized the factors influencing the quality and evaluation process of restorations. The first group of factors are restoration related (material type, composition, insertion technique, curing mode, finishing and polishing, shading concept, and accuracy). The second group is preparation related (form and configuration, volume, margin design, and proximal extension). The third group is operator related (experience, skills, and handling). The environment in terms of public academic centers, private centers, public social centers, multiple or single operator practice was listed as the fourth group. ^7^^,^^32^^,^^33^

In designing the questionnaire of the current study and the targeted population, the previous factors were considered.

Modern resin composite formulations include developments and modifications in materials technology regarding the structural components of resin matrix, fillers, coupling agents, photo initiator as well as shading and color pigments. ^34^^–^^38^ This has greatly influenced the physico-mechanical and biological properties of different brands of resin composites which eventually impact clinical application techniques and final clinical behavior during function in the oral cavity. Bulk-fill, self-adhesive, single shade, self-healing, and antibacterial composites are examples of such recent developments. Similarly, modern finishing and polishing tools of resin composites have also become available in the market. The increasing demand from patients for excellent esthetic quality has been met with distinctive clinical application techniques utilizing direct resin composites for restoring defects of particularly anterior teeth, this by means of combining art with knowledge and color selection. ^5^ Based on the natural layering concept, a polychromatic layering technique has been suggested. ^39^ An example of this practice is the utilization of palatal indices to optimize the designed form and contours. ^40^

Isolation of the operative field and control of moisture during various operative restorative procedures are essential for developing optimum physical and mechanical properties of the restoration. This is particularly true for resin composite restorations in anterior teeth where developing optimum adhesion and superior esthetics is mandatory for clinical reliability and patient satisfaction. In agreement with previous studies, rubber dam followed by cotton rolls isolation were the two methods of operative field isolation used by participants of the current survey. ^41^

Assessing the ability to reliably restore esthetics is a principal aspect of the current cross-sectional study. The chameleon effect has been reported by manufacturers of resin composites as a property of the material to adapt to the color of the surrounding tooth tissues. The color adjustment potentials of resin composites have been reported to be influenced by the material type, the cavity design, and configurations as well as the substrate surrounding the restoration. Understanding the optical characteristics of teeth and properties of incident light is essential for optimum shade selection and faithful restoration of esthetic derangements. ^42^ Making use of the optical illusion phenomenon is essential for optimum esthetic outcomes and excellence in restoring anterior tooth form, contour, and emergence profile. Accordingly, the best esthetic outcomes are achievable when scientific knowledge is coupled with artistic potential. ^43^ Consequently, dentists should be continuously updated in materials and color selection technologies so as to be able to select and implement the best application technique for each clinical indication. Selection of the most appropriate brand of resin composite material, adhesive approach and insertion technique for a specific clinical case is crucial for a clinically satisfactory result, particularly when restoring deranged esthetics in anterior tooth defects. This should be based on thorough knowledge of color dimensions, optical properties, principles, and technologies of shade selection, ^44^ familiarity of the respective material properties, indications and contraindications, comprehensive esthetic analysis including tooth forms, alignment, symmetry, the correlation with adjacent and opposing teeth, ^45^ and in addition, the clinical factors related to the specific lesion or tooth defect. ^46^ Moreover, full recognition of complex oral environmental factors influencing decision making in the designing of treatment plans has a major influence on the quality and clinical reliability of the final anterior resin composite restoration and is mandatory for assuring patient satisfaction. ^17^^,^^18^^,^^47^^,^^48^ The third domain of our questionnaire therefore aimed to study the awareness of general dentists concerning the different composite classes of material and the respective clinical indications. Participants were asked about class V resin composite restorations for restoring cervical tooth defects including abfraction lesions. These restorations are used to restore cervical tooth defects where the greatest flexure of teeth occurs during functioning, and includes higher risks of developing tooth cracking, abfraction defects and loss of retention with the usage of rigid and higher inorganic filler containing restorations. Accordingly, the need for more flexible and less filler containing restorations that yield upon repeated flexure at cervical locations, including having superior surface finish ability and polish ability, is clearly advocated in these cases. Our results showed that for class V lesions, 28% of participants use microfilled resin composites while 28% apply nanohybrid composites, 26% use flowable composites and 16% insert micro-hybrid composite, raising strong concerns.

Because of their initial low viscosity and high post gel flexibility, many previous studies have recommended the use of flowable composites for restoring non-carious cervical tooth defects. ^49^ Nevertheless, our collected data indicated that in class III restorations, 37 % work with nano-hybrid, and 28% of participants use micro-filled, and 32% use micro-hybrid composites. In a randomized clinical control trial over 12 months, ^50^ micro-hybrid resin composite was found to provide the best color match over microfilled and nanohybrid composite while microfilled and nanofilled composite showed better 12-month surface quality than microhybrid composite.

Our results for class IV restorations where high strength resin composite materials are highly recommended, showed that 33% of the respondents employ nano-hybrid while another 33% use micro-hybrid resin composites, and 26% used microfilled composite, 16% use other resin composite classes, and 2% of non-respondents, deserves attention. In a comprehensive metanalysis, ^51^ the main reason for replacement of class IV resin composites was bulk fracture which occurred more frequently with microfilled composite than with hybrid and macrofilled composites.

The literature shows that there is no one single class of resin composite for all purposes and that it is the responsibility of the restorative dentist to select the most appropriate type of composite for a particular indication. In response to the current questionnaire, many dentists indicated that selection of composite material conforms to sound and relevant evidence-based criteria for selection in respective clinical situations. On the other hand, other responses indicated unjustified selections with poor clinical relevance and judgement.

The survey indicated a clear variation between respondents regarding the specific adhesive regularly used. The easier and less time-consuming usage of self-etching adhesives have apparently played a role in individual operator preference at the expense of the proven long-term clinical reliability and more consistent performance of total-etch adhesives. This might explain that only 45% of participants use total-etch adhesives, while 31% and 23% used self-etching and selective etching technology, respectively. This is in line with a similar study ^3^ finding.

Presently, most resin-based composites are visible light-activated materials. This allows for more controlled and command curing and adequate working time before curing, while at the same time requiring incremental placement of material into the prepared cavity to ensure an optimum degree of curing. ^52^^–^^54^ Adequate light curing is crucial to achieve the desired biomechanical and biocompatibility properties of the resin composite material. Adequate curing requires appropriate criteria of light intensity, curing time, direction of the curing light and proximity of the light curing source to the surface of the curing resin composite. ^55^^–^^57^ Dental light-curing units are handheld devices that are used for the polymerization of visible light-activated dental materials. Different light-curing units of variable technologies are currently available in the market. Each has specific set-up and light intensity output. Respondents showed variation in their practice of light curing and specific curing devices. ^58^ It is evident that light curing awareness and practices need urgent reconsideration and correction in many cases. LED followed by QTH are the most widely used light curing devices while Laser and Plasma Arch are the least used light curing devices. This is in line with many pervious reports indicating the popularity of LED and QTHs. ^59^ A previous study found LED units to have higher curing light intensity than that of QTH. Moreover, it reported that QTHs have greater frequency than LEDs. ^60^ Due to the higher radiant emmitance of plasma arch light and argon ion laser, less light curing time is needed. However, greater polymerization shrinkage stresses and marginal discrepancies were reported with this mode of light curing. ^58^

Previous reports have advocated that in anterior teeth with extensive loss of coronal tooth structure involving the incisal edge, the controlled application of resin composite restorations using a palatal putty consistency rubber base index is more recommended than the freehand application. Cumulating evidence indicates that the palatal index technique allows multiple chromatic layering, leading to improved tooth optical characteristics in addition to optimizing the restoration of tooth form and contours with a minimal need for occlusal adjustment. ^61^^–^^64^ The results of the current survey indicated that 46% of participants used both techniques while only 10% exclusively used the palatal index technique. This contradicts evidence-based research and therefore reflects the need for practice correction. Moreover, the survey indicated that many dental practitioners in Saudi Arabia should consider changing their practice from using single shades of resin composite to the polychromatic approach of applying several shades of resin composite when restoring anterior tooth defects, so as to achieve the best esthetic quality. A previous report described the multiple layering approach using different enamel and dentin composite shades, a palatal hybrid composite layer, and a buccal microfilled composite with selective addition of transparent and white spot characters as a biomimetic direct composite stratification technique duplicating the physical and optical properties of natural teeth in class IV restorations. ^65^

Finishing and polishing of resin composites are essential for long-term clinical reliability and esthetically pleasing restorations. Smooth surfaces will not accumulate plaque and stains with subsequent discoloration and secondary decay. ^66^ Respondents commonly selected various tools in finishing and polishing resin composite rather than using one single tool for finishing and polishing, thereby reflecting the general keenness of respondents to perform optimum finishing and polishing procedures. Finishing and polishing strips were the preferred tools for 69% of respondents that might be related to their ease of usage, especially with class III restorations. Participants also reported other tools like white stones, Soflex, and impregnated rubber tips and polishing paste as other validated options which are in line with previous studies. ^67^^–^^69^ The current survey responses from dentists in Saudi Arabia showed that discoloration is most common form of failure and patient dissatisfaction of resin composites in anterior teeth, which is generally in line with previous outcomes of systematic reviews listing reasons for failure of resin composite restorations. ^70^ Accordingly, upon restoring anterior tooth defects with resin composites, clinical practices of dentists in Saudi Arabia should confirm superior long-term pleasing esthetics and minimization of future caries recurrence. Furthermore, it is recommended that dental practitioners in Saudi Arabia implement clinical protocols that will prevent or minimize post-restoration hypersensitivity, ensure best tooth form, and provide durable structural integrity without chipping or fracture of the restored anterior tooth. The current survey outcomes have also identified the need for dental practitioners in Saudi Arabia to upgrade their awareness and to modify their practices of resin composite restorations in anterior tooth defects. Participation in continuous dental educational and training programs is strongly suggested. Improving the clinical skills and broadening the experiences of undergraduates and recent graduates in complex esthetic resin composite restorative techniques under close supervision of senior clinical experts is vital. ^71^

## Conclusions

In consideration and within the limitations of this study, the following conclusions can be made:

1- Dental practitioners in Saudi Arabia are not consistent in their clinical practice of restoring anterior tooth defects using resin composites, regardless of the location of their practice or length of their clinical experience.
2- Improvement in clinical practice and awareness of resin composite restorations in anterior teeth appears to be of high necessity for some dental practitioners in Saudi Arabia. Dental practitioners should actively and dynamically be familiar with updates in resin composites technologies and clinical techniques of restoring anterior teeth through continuous education programs, workshops, and online webinars.

## Data Availability

Zenodo: Toward esthetically and biomechanically reliable anterior resin composite restorations: current clinical experiences among dental practitioners in Saudi Arabia. https://doi.org/10.5281/zenodo.7738566. This project contains the following underlying data:
- Responses collected-Anterior Composite.xlsx Data are available under the terms of the Creative Commons Attribution 4.0 International license (CC-BY 4.0).
