# Supplementary material for: Toward esthetically and biomechanically reliable anterior resin composite restorations: Current clinical experiences among dental practitioners in Saudi Arabia
Source: F1000Res. 2023 Sep 20;12:359. Originally published 2023 Apr 3. [Version 2] doi: 10.12688/f1000research.130981.2 (PMC10556563; doi:10.12688/f1000research.130981.2)
Supplement: Peer review history

# Reviewer report: Reviewer response for version 2

**DOI:** 10.5256/f1000research.155843.r208137

**Published:** 2023-10-05

**Authors:** Hatem M. El-Damanhoury

**Affiliations:** 1 Department of Preventive and Restorative Dentistry, University of Sharjah, Sharjah, United Arab Emirates

The authors addressed all the reviewer's comments, and no further changes are required.

Is the work clearly and accurately presented and does it cite the current literature?

Yes

If applicable, is the statistical analysis and its interpretation appropriate?

Yes

Are all the source data underlying the results available to ensure full reproducibility?

Yes

Is the study design appropriate and is the work technically sound?

Yes

Are the conclusions drawn adequately supported by the results?

Yes

Are sufficient details of methods and analysis provided to allow replication by others?

Yes

Reviewer Expertise:

Restorative Dentistry

I confirm that I have read this submission and believe that I have an appropriate level of expertise to confirm that it is of an acceptable scientific standard.

# Reviewer report: Reviewer response for version 2

**DOI:** 10.5256/f1000research.155843.r208136

**Published:** 2023-09-29

**Authors:** Nesrine A. Elsahn

**Affiliations:** 1 Operative Dentistry, Cairo University, Giza, Giza Governorate, Egypt; 2 Department of Clinical Sciences, College of Dentistry, Ajman University, Ajman, United Arab Emirates

I have no further comments to make.

Is the work clearly and accurately presented and does it cite the current literature?

Yes

If applicable, is the statistical analysis and its interpretation appropriate?

Yes

Are all the source data underlying the results available to ensure full reproducibility?

Yes

Is the study design appropriate and is the work technically sound?

Yes

Are the conclusions drawn adequately supported by the results?

Yes

Are sufficient details of methods and analysis provided to allow replication by others?

Yes

Reviewer Expertise:

Adhesive Dentistry, Restorative Dentistry, Dental Materials.

I confirm that I have read this submission and believe that I have an appropriate level of expertise to confirm that it is of an acceptable scientific standard.

# Reviewer report: Reviewer response for version 1

**DOI:** 10.5256/f1000research.143783.r186405

**Published:** 2023-08-10

**Authors:** Nesrine A. Elsahn

**Affiliations:** 1 Operative Dentistry, Cairo University, Giza, Giza Governorate, Egypt; 2 Department of Clinical Sciences, College of Dentistry, Ajman University, Ajman, United Arab Emirates

The article presents a comprehensive and well-executed study that investigates the awareness of dental practitioners in Saudi Arabia about resin composite restorations in anterior teeth. The authors have clearly outlined the research problem, framed it within the relevant literature and the methodology employed is appropriate for addressing the research questions posed. Moreover, the study provided evidence-based recommendations to improve anterior restorations in Saudi Arabia. However, the authors need to address the following points and answer the following questions. 

Comment #1: The highlighted parts in the following paragraphs contain information that was stated twice. The authors have to revise and rephrase these parts to avoid repetition.

 "This cross-sectional study was conducted utilizing an online uploaded survey questionnaire using Google’s free online survey platform. **E-mail invitations stated clearly that participation was anonymous and voluntary and that participants were free to leave the survey or to skip any questions.** The targeted population of 500 resulted in a calculated sample size of 218 dentists. Calculations were performed at the 95% confidence level using a free online sample size calculator."

 "During the research, **participants were invited to join the survey through emails that clarified the objectives of the study and included necessary information and that joining the survey is anonymous and voluntary. The invite explained that by completing the survey, they were consenting to participate in the study. Participants were informed that they could leave the study at any step if they did not wish to continue**, and that the data collected would be used for research purposes."

Comment #2: Why was the Nanofilled composite not listed as an option to restore different classes of anterior tooth defects?

Comment #3: In Domain 2:

1. There is no table number or legend/ header for the information related to questions 5 and 6. If the authors decided to present this part in a table, a table number must be given and all the changes required in the following tables’ numbering and in the text must be done.
2. The sample size (n=250) has to be written before the table for questions 5 and 6, as it was done for the other tables, not at the end of the questions.
3. The authors didn’t include answer options, participants’ responses, or relative responses in percentages for question #6. Although this information was presented in Figure 2, the authors have either to present domain 2 in figures only or to use figures and a table, provided that the table is written following the same format used for the other tables.

Comment #4: In question 8, what does option build-up mean or represent? Was it clarified for the participants?

Comment #5: In question 10, the authors mentioned in the discussion that only 45% of participants use total-etch adhesives, while 31% and 23% used self-etching and selective etching technology, respectively.

 The authors used the correct terminology for the adhesive strategies in the discussion. Meanwhile, in Table 3 question 10, the authors used a different terminology for the adhesive types (self-adhesive, selective self-etching, total-etching). The answer options for question 10 need to be replaced by the correct terminology for adhesive strategies. Otherwise, the authors need to clarify in the text the adhesive strategies' terms they used.

Comment# 6: Why the statistical analysis was done only for domains 3 and 4?

Comment #7: The authors used reference 65 to describe the multiple-layering approach. Please add a recent reference that introduces the latest techniques and composite materials used in the multiple-layering approach.

Is the work clearly and accurately presented and does it cite the current literature?

Yes

If applicable, is the statistical analysis and its interpretation appropriate?

Yes

Are all the source data underlying the results available to ensure full reproducibility?

Yes

Is the study design appropriate and is the work technically sound?

Yes

Are the conclusions drawn adequately supported by the results?

Yes

Are sufficient details of methods and analysis provided to allow replication by others?

Yes

Reviewer Expertise:

Adhesive Dentistry, Restorative Dentistry, Dental Materials.

I confirm that I have read this submission and believe that I have an appropriate level of expertise to confirm that it is of an acceptable scientific standard, however I have significant reservations, as outlined above.

# Reviewer report: Reviewer response for version 1

**DOI:** 10.5256/f1000research.143783.r186404

**Published:** 2023-07-28

**Authors:** Hatem M. El-Damanhoury

**Affiliations:** 1 Department of Preventive and Restorative Dentistry, University of Sharjah, Sharjah, United Arab Emirates

This is a well-written manuscript, that investigated the awareness of general dental practitioners in Saudi Arabia about resin composite restorations in anterior teeth using a cross-sectional survey study design. 

 The sample size and methodology is appropriate, the statistical analysis is suitable for the data type and the presentation of the results is adequate to draw conclusions.

 A few points have to be addressed by the authors before accepting this manuscript for indexing:

- More details of the sample size calculation method are required. 
- For the validity and reliability of the questions, it is recommended to run an inter-examiner agreement test.
- Please explain why the Chi-square test was used and whether the assumption of the test was met or not.
- Please delete the sentence "Minitab statistical software is an example of free online software that can be used to perform similar statistical analysis", as it is not needed, and adds nothing to the manuscript.

Is the work clearly and accurately presented and does it cite the current literature?

Yes

If applicable, is the statistical analysis and its interpretation appropriate?

Yes

Are all the source data underlying the results available to ensure full reproducibility?

Yes

Is the study design appropriate and is the work technically sound?

Yes

Are the conclusions drawn adequately supported by the results?

Yes

Are sufficient details of methods and analysis provided to allow replication by others?

Yes

Reviewer Expertise:

Restorative Dentistry

I confirm that I have read this submission and believe that I have an appropriate level of expertise to confirm that it is of an acceptable scientific standard, however I have significant reservations, as outlined above.
